# Noninvasive assessment of liver fibrosis with aspartate transaminase to platelet ratio index (APRI)

**Published:** 2011-07-01

**Authors:** Michael Adler

**Affiliations:** 1 Department of Gastroenterology, Hepato-Pancreatology and Digestive Oncology, Erasmus Hospital Brussels, Brussels, Belgium

**Keywords:** Liver diseases, Cirrhosis, Chronic hepatitis

Dear Editor,

In chronic liver diseases, the management of patients must include a determination of the stage of fibrosis (to select specific therapies), a prognosis, the prevention of complications, and the surveillance of the disease. Over the past several years, significant progress has been made in improving noninvasive methods of assessing liver fibrosis. The risks of liver biopsy and the potential for sampling errors with regard to fibrosis staging support the use of noninvasive modalities including serum fibrosis markers or scores and elastography. The first are classified as direct (representing components of the extracellular matrix) or indirect (reflecting hepatic inflammation and function) and included in panels for clinical use. They include patented (i.e., Fibrotest, Fibrometer) and nonpatented (ASL/ALT ratio, APRI, FIB-4, Forns, ELF, Hepascore) tests. The majority of studies has involved patients with chronic HCV infection. Direct and indirect methods have demonstrated good to excellent performance in detecting significant disease (≥ F2) and cirrhosis (F4) [1]. Transient elastography (Fibroscan®), which measures liver stiffness, has excellent accuracy in detecting cirrhosis (F4) in chronic liver diseases [2]. The APRI (AST-to-platelet count ratio) is the most simple and cheapest indirect marker of inflammation and fibrosis [3]. Its diagnostic performance in detecting advanced fibrosis has been evaluated extensively [4], showing low sensitivity (41%), low negative predictive values (64%), good specificity (95%) and high positive predictive values (88%).

In a study, a group of Turkish investigators presented their experience with the APRI in patients with chronic liver disease [5]. In a retrospective series of 455 patients (207 with HBV, 108 with HCV, and 140 with NAFLD) the low value [1] median Metavir fibrosis score with median values for APRI were reported 0.46, 0.49 and 0.43 respectively in the HBV, HCV and NAFLD groups. AUROC values for the detection of fibrosis (1 to 4) versus no fibrosis (F0) were 0.58, 0.54, and 0.62, respectively, in the 3 groups. Dr. Yilmaz and his team concluded that the APRI has acceptable accuracy in assessing liver fibrosis in patients with HCV and NAFLD but not in those with HBV. There are several drawbacks and flaws in this report that render its message unrealistic and erroneous. It was a retrospective series, without validation in an independent series, with 3 categories of etiologies, each comprising a limited number of patients. It was not a consecutive series, and the indication for liver biopsy was not mentioned, preventing the results from being applicable to other clinicians throughout the world. Furthermore, it is difficult to understand the basis for the diagnosis of NAFLD: US detection of steatosis ≥ 1 and absence of other causes of liver disease.

The main weaknesses of this study were that a poor marker was chosen and the use of a non-pertinent clinical endpoint (i.e., presence or of fibrosis not) in place of significant fibrosis (≥ F2) and cirrhosis (≥ F4) or advanced (≥ F3) fibrosis. Thus, it is not surprising that the APRI performed poorly, with AUROCs largely inferior to the minimal value of 80%, well accepted by the medical community [6]. Even Dr. Alberti's group in Italy [7], which has great experience with the APRI, admits that this test alone has poor and variable performance, even in the identification of cirrhosis (AUROCs from 0.61 to 0.94 and 0.69 to 0.88 for significant [i.e. ≥ F2] fibrosis) when used alone. They propose a model, called "Sequential Algorithm for Fibrosis Evaluation = safe biopsy algorithm," in which APRI is used first, after which Fibrotest® is used as the second-line test in the setting of HCV and HBV, effecting 47% and 82% spared liver biopsies in significant fibrosis and cirrhosis, respectively. Our group also has experience with the APRI, and we sought to compare, independently from the promoters, its diagnostic accuracy using AUROCs for the prediction of significant, advanced, and cirrhosis in HCV and other etiologies. Fibrotest was the most effective, followed by FIB-4, FORNS, APRI, and Fibroindex, in order of decreasing accuracy. In the global series and the HCV series, the AUROCs of the APRI were 0.73 and 0.74 for the diagnosis of significant fibrosis, reinforcing the observation that the minimal cut-off of 80% was not reached.

In conclusion, the APRI alone is inappropriate for use in assessing liver fibrosis.
